# *GREB1* ‐rearranged uterine tumour shares a common DNA methylation signature with *ESR1* ‐rearranged UTROSCT

**DOI:** 10.1111/his.70075

**Published:** 2025-12-22

**Authors:** Cheng‐Han Lee, Yow‐Shan Lee, Jennifer A Bennett, David L Kolin, Jen‐Chieh Lee, Hsuan‐Ying Huang, Martin Köbel, Mark Sementsov, Brendan C Dickson, Christian Koelsche, Friedrich Kommoss, Andreas von Deimling, Felix K F Kommoss

**Affiliations:** ^1^ Department of Laboratory Medicine and Pathology University of Alberta Edmonton Alberta Canada; ^2^ Department of Family Medicine Cathay General Hospital Taipei Taiwan; ^3^ University of Chicago Medical Center Chicago Illinois USA; ^4^ Division of Women's and Perinatal Pathology, Department of Pathology Brigham and Women's Hospital Boston Massachusetts USA; ^5^ Department of Pathology National Taiwan University Hospital, National Taiwan University College of Medicine Taipei Taiwan; ^6^ Department of Anatomic Pathology Kaohsiung Chang Gung Memorial Hospital and Chang Gung University College of Medicine Kaohsiung Taiwan; ^7^ Department of Pathology and Laboratory Medicine University of Calgary Calgary Alberta Canada; ^8^ Institute of Pathology, Heidelberg University Hospital Heidelberg Germany; ^9^ Department of Pathology and Laboratory Medicine Sinai Health System and University of Toronto Toronto Ontario Canada; ^10^ Institute of Pathology, Ludwig Maximilians University Hospital Munich Munich Germany; ^11^ Institute of Pathology, Medizin Campus Bodensee Friedrichshafen Germany; ^12^ Department of Neuropathology Heidelberg University Hospital Heidelberg Germany; ^13^ Clinical Cooperation Unit Neuropathology, DKFZ Heidelberg Germany

**Keywords:** DNA methylation, ESR1, GREB1, uterine sarcoma, UTROSCT

## Abstract

**Background and objectives:**

*GREB1*‐rearranged uterine tumours encompass a group of uterine mesenchymal tumours with varied histologic appearances. The fusion partners to *GREB1* include *NCOA1‐3, SS18* and *NR4A3*. Given that some *GREB1*‐rearranged uterine tumours exhibit histologic features of uterine tumours resembling ovarian sex cord tumour (UTROSCT), there is a general belief that *GREB1*‐rearranged uterine mesenchymal tumours are part of the UTROSCT family.

**Methods:**

In this study, we applied global DNA methylation and copy number analyses to a series of 10 *GREB1‐*rearranged uterine tumours and 21 classic UTROSCTs (7 of which were molecularly confirmed to harbour *ESR1::NCOA2/3* fusions).

**Results:**

We found that *GREB1*‐rearranged uterine tumors show an overlap in their global methylation profiles with UTROSCT, including *ESR1::NCOA2/3* positive cases. Together, these tumours form a DNA methylation cluster separate from uterine smooth muscle tumours (leiomyomas and leiomyosarcomas), endometrial stromal sarcomas (low‐grade and high‐grade), embryonal rhabdomyosarcoma and SMARCA4‐deficient uterine sarcomas. However, despite their epigenetic similarity, there were two notable differences. First, *GREB1*‐rearranged uterine tumours as a group displayed a greater degree of genomic complexity with more extensive copy number alterations than conventional UTROSCTs, including those harbouring *ESR1::NCOA2/3*. Second, *GREB1*‐rearranged uterine tumours frequently lacked overt sex cord morphology: while all 7 *ESR1::NCOA2/3* UTROSCTs demonstrated corded, nested, trabecular and/or tubular/sertoliform patterns, only 1 *GREB1*‐rearranged uterine tumour displayed a prominent trabecular pattern, with the remaining cases showing exclusively or predominantly diffuse/solid growth.

**Conclusions:**

Overall, our findings confirm that *GREB1*‐rearranged uterine tumours are part of the UTROSCT spectrum, though they frequently exhibit a more diffuse growth pattern and a higher degree of genomic instability.

## Graphical abstract

GREB1‐rearranged uterine tumours share DNA methylation profiles with UTROSCTs, supporting a close relationship. Despite differences in morphology and genomic complexity, their epigenetic similarity supports the inclusion of these tumours within the UTROSCT spectrum.

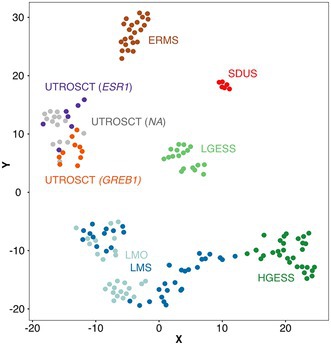

**Abbreviations**
FFPE: formalin‐fixed paraffin‐embedded
FISH: fluorescence in‐situ hybridization
GI: Genomic Index
H&E: haematoxylin and eosin
t‐SNE: t‐distributed stochastic neighbour embedding
UTROSCT: uterine tumours resembling ovarian sex cord tumour

## Introduction

Uterine tumour resembling ovarian sex cord tumour (UTROSCT) is a uterine neoplasm of low malignant potential with a reported recurrence rate that ranges from 6% to 23%.^1^, ^2^, ^3^ UTROSCT was initially described by Clement and Scully in 1976. In their original paper, the authors separated UTROSCT into two groups, with group 1 tumours comprising endometrial stromal tumours with focal sex cord differentiation and group 2 tumours comprising tumours with exclusive differentiation reminiscent of an ovarian sex cord tumour.^4^ Molecular insights subsequently confirmed that tumours displaying group 1 features represent endometrial stromal tumours with sex cord differentiation, as they harbour genetic fusions that are specific to endometrial stromal neoplasms.^5^, ^6^, ^7^, ^8^ As such, the current WHO tumour classification defines UTROSCT as a uterine neoplasm with morphological patterns that resemble those seen in ovarian sex cord tumours, without a component of recognizable endometrial stromal neoplasia.^9^ In early 2019, Dickson *et al*. described 3 UTROSCT harbouring *ESR1::NCOA2/3* fusions and one case with a *GREB1::NCOA2* fusion, while Croce *et al*. described a UTROSCT harbouring a *GREB1::CTNNB1* fusion.^10^, ^11^ All 5 UTROSCTs contained areas of morphologically apparent sex cord differentiation, particularly with anastomosing tubular/corded/trabecular histology. Notably, the UTROSCT harbouring the *GREB1::NCOA2* fusion was comprised predominantly of spindle cell fascicles with a minor component of interspersed tubules, and the UTROSCT harbouring *GREB1::CTNNB1* was associated with extrauterine metastasis. Later in 2019, we described a series of uterine tumours with minimal sex cord differentiation harbouring *GREB1* fusions (*GREB1::NCOA1*, *GREB1::NCOA2*, *GREB1::NR4A3* and *GREB1::SS18*). Our findings suggested that tumours harbouring *GREB1* fusions more frequently display inconspicuous sex cord differentiation, tend to be larger in size, occur in older women and may behave more aggressively than *ESR1*‐rearranged UTROSCT.^12^ In 2020, Goebel *et al*. reported on the pathologic and molecular features of 26 morphologically classic UTROSCTs and 82% of their cases demonstrated evidence of genetic fusion between *ESR1* or *GREB1* and *NCOA1/2/3*, indicating histologic overlap between *GREB1*‐rearranged and *ESR1*‐rearranged UTROSCTs.^13^ More recently, Bi *et al*. described 23 molecularly defined UTROSCTs that included 12 *GREB1*‐rearranged and 10 *ESR1*‐rearranged UTROSCTs, which mirrored our findings that patients with *GREB1‐*rearranged tumours were older, had larger tumours and higher stage than patients with non‐*GREB1*‐rearranged tumours.^14^ Interestingly, 6 of the 12 *GREB1*‐rearranged UTROSCTs were misdiagnosed as another tumour type, whereas only 1 of the 10 *ESR1*‐rearranged UTROSCTs was misdiagnosed initially, which suggests that sex cord differentiation in *GREB1*‐rearranged UTROSCTs may be less conspicuous. In a recent systemic review by Maccio *et al*. that included 88 molecularly classified UTROSCTs, they found that *GREB1*‐rearranged UTROSCTs were associated with older age, larger tumour size, higher mitotic index, more frequent lymphovascular invasion and significantly lower disease‐free survival compared to *ESR1*‐rearranged UTROSCTs.^15^

Recently, DNA methylation‐based classification of tumours has emerged as a reliable tool for delineating tumour entities, and we have previously demonstrated that uterine tumours, including morphologically classic UTROSCTs, are characterized by distinct DNA methylation profiles.^16^ Here we analysed a cohort of UTROSCTs, including tumours harbouring *ESR1* and *GREB1* rearrangements by array‐based DNA methylation analysis, to gain further insights into the nosology and biology of these seemingly related but also somewhat different tumours.

## Material and Methods

### Study Cohort

We collected a multicentre cohort of 21 UTROSCT from the reference files of the authors of this study. A subset of these tumours had previously been analysed by next‐generation sequencing or fluorescence in‐situ hybridization (FISH) at the contributing institutions. Additionally, we collected a series of 10 uterine tumours harbouring *GREB1* rearrangements, 7 of which were previously reported in earlier publications.^10^, ^12^ All available haematoxylin and eosin (H&E) stained slides from formalin‐fixed paraffin‐embedded (FFPE) tissue samples were reviewed (range 1–12). Medical records were reviewed for clinical data. An ANOVA followed by Tukey's HSD post‐hoc test was used to assess statistically significant differences between groups.

### DNA Extraction and Array‐Based Analysis

DNA was extracted from FFPE tumour tissue using the Maxwell® 16 FFPE Plus LEV DNA Kit or the Maxwell® 16 Tissue DNA Purification Kit (for frozen tissue) on the automated Maxwell device (Promega, Madison, WI, USA) according to the manufacturer's instructions. A minimum of 100 ng of DNA was subjected to bisulphite conversion and processed on the Illumina Infinium EPIC (850 k) BeadChip (Illumina, San Diego, USA) according to the manufacturer's instructions.

### DNA Methylation Analysis

DNA methylation data analysis was performed in R using packages from Bioconductor.^17^ Data were normalized using background correction and dye bias correction (shifting the mean intensity of negative control probes to zero and scaling the mean intensity of normalization control probes to 20,000). Probes targeting sex chromosomes, those containing multiple single‐nucleotide polymorphisms, and probes that could not be uniquely mapped were removed. For subsequent DNA methylation analyses, we included a previously compiled methylation dataset of a large cohort of various uterine sarcomas with different gene fusions, including low‐grade endometrial stromal sarcoma (LGESS; *n* = 18) with *JAZF1::SUZ12*, *JAZF1::PHF1*, *EPC1::PHF1* and *MEAF6::PHF1* gene fusions, high‐grade endometrial stromal sarcoma (HGESS; *n* = 31) with *YWHAE::NUTM2A/B*, *ZC3H7B::BCOR*, *BCORL1* rearrangements and *BCOR* ITD, as well as leiomyomas (LMO; n = 27), leiomyosarcomas (LMS; *n* = 37), *DICER1*‐mutant embryonal rhabdomyosarcomas (ERMS; *n* = 22) and *SMARCA4*‐deficient uterine sarcomas (SDUS; *n* = 6).^16^, ^18^, ^19^, ^20^ For unsupervised hierarchical clustering of DNA methylation data, 10,000 probes with the most variably methylated probes across the dataset were selected. Distance between samples was calculated using Euclidean distance, and average linkage was used to generate dendrograms. For an unsupervised 2D representation of pairwise sample correlations, dimensionality reduction was performed using t‐distributed stochastic neighbour embedding (t‐SNE) with the 10,000 most variable probes, a perplexity of 10 and 3000 iterations. The stability of methylation groups was tested by varying the number of the most variable probes.

### Copy Number Analysis

CNV analysis was performed by analysing DNA methylation array data using the R‐package *copynumber*.^21^ Gene amplifications and deletions were detected by manual inspection of CNV profiles. The Genomic Index (GI), indicative of genomic complexity, was calculated as previously described (total number of segmental gains or losses^2^/number of involved chromosomes).^22^ The upper and lower thresholds for segmental gains and losses were set at 0.1 and −0.1 (log2), respectively.

## Results

### Study Cohort

The study cohort consisted of 21 classic UTROSCT defined by characteristic morphologic features, of which 14 were not tested for gene rearrangements and 7 harboured *ESR1* rearrangements (*ESR1::NCOA2*, *n* = 4; *ESR1::NCOA3*, *n* = 3). For comparison, the study cohort included 10 uterine tumours harbouring *GREB1* rearrangements (*GREB1::NCOA1*, *n* = 2; *GREB1::NCOA2*, *n* = 3; *GREB1::NCOA3*, *n* = 1; *GREB1::NR4A3*, *n* = 1; *GREB1::SS18*, *n* = 1; *GREB1* rearrangement identified by *GREB1* break apart FISH, *n* = 2). In our cohort, patients with *GREB1*‐rearranged tumours were older (mean 59 years; median 58 years) than those with *ESR1‐*rearranged tumours (mean 47 years; median 46 years) or tumours without molecular testing (mean 44.6 years; median 42.5 years). However, this difference was not statistically significant (*P* = 0.06). Clinicopathological and molecular characteristics of the study cohort are summarized in Table 1.

**Table 1:** Clinicopathological and molecular characteristics of the study cohort (*n* = 31)

Study ID	Age at diagnosis	Alteration	Genomic index	Mitoses per 2 mm^2^	Sex cord elements	Sex cord pattern
1	32	Not tested	1	<1	Yes	Solid and corded
2	61	Not tested	1	1	Yes	Trabecular, sertoliform and solid
3	63	Not tested	2	<1	Yes	Trabecular, nested and sertoliform
4	51	Not tested	2	<1	Yes	Corded, nested and solid
5	40	Not tested	3	2	Yes	Solid and sertoliform
6	56	Not tested	3	<1	Yes	Sertoliform
7	40	Not tested	3	<1	Yes	Sertoliform
8	26	Not tested	4	<1	Yes	Sertoliform
9	45	Not tested	5	2	Yes	Solid and corded
10	26	Not tested	5	<1	Yes	Solid and corded
11	36	Not tested	6	<1	Yes	Sertoliform
12	67	Not tested	7	1	Yes	Trabecular, nested and sertoliform
13	32	Not tested	7	<1	Yes	Solid and corded
14	50	Not tested	12	<1	Yes	Corded
15	46	*ESR1::NCOA2*	2	1	Yes	Solid, nested and corded
16	75	*ESR1::NCOA2*	4	<1	No	Solid
17	26	*ESR1::NCOA2*	8	2	Yes	Sertoliform and trabecular
18	66	*ESR1::NCOA2*	20	<1	Yes	Solid and corded
19	23	*ESR1::NCOA3*	6	1	Yes	Solid and corded
20	40	*ESR1::NCOA3*	16	2	Yes	Sertoliform
21	53	*ESR1::NCOA3*	18	1	Yes	Sertoliform and trabecular
22	49	*GREB1* rearrangement	4	2	No	Solid
23	53	*GREB1* rearrangement	40	<1	No	Solid
24	81	*GREB1::NCOA1*	24	2	No	Solid
25	69	*GREB1::NCOA1*	29	9	No	Solid
26	61	*GREB1::NCOA2*	12	1	Yes	Solid with very minor sertoliform
27	56	*GREB1::NCOA2*	14	<1	No	Solid
28	56	*GREB1::NCOA2*	29	13	No	Solid
29	37	*GREB1::NCOA3*	2	<1	No	Solid
30	60	*GREB1::NR4A3*	32	14	Yes	Trabecular and minor solid
31	68	*GREB1::SS18*	14	4	No	Solid

### Histologic Features of ESR1‐Rearranged and GREB1‐Rearranged UTROSCTs

**Figure 1:**
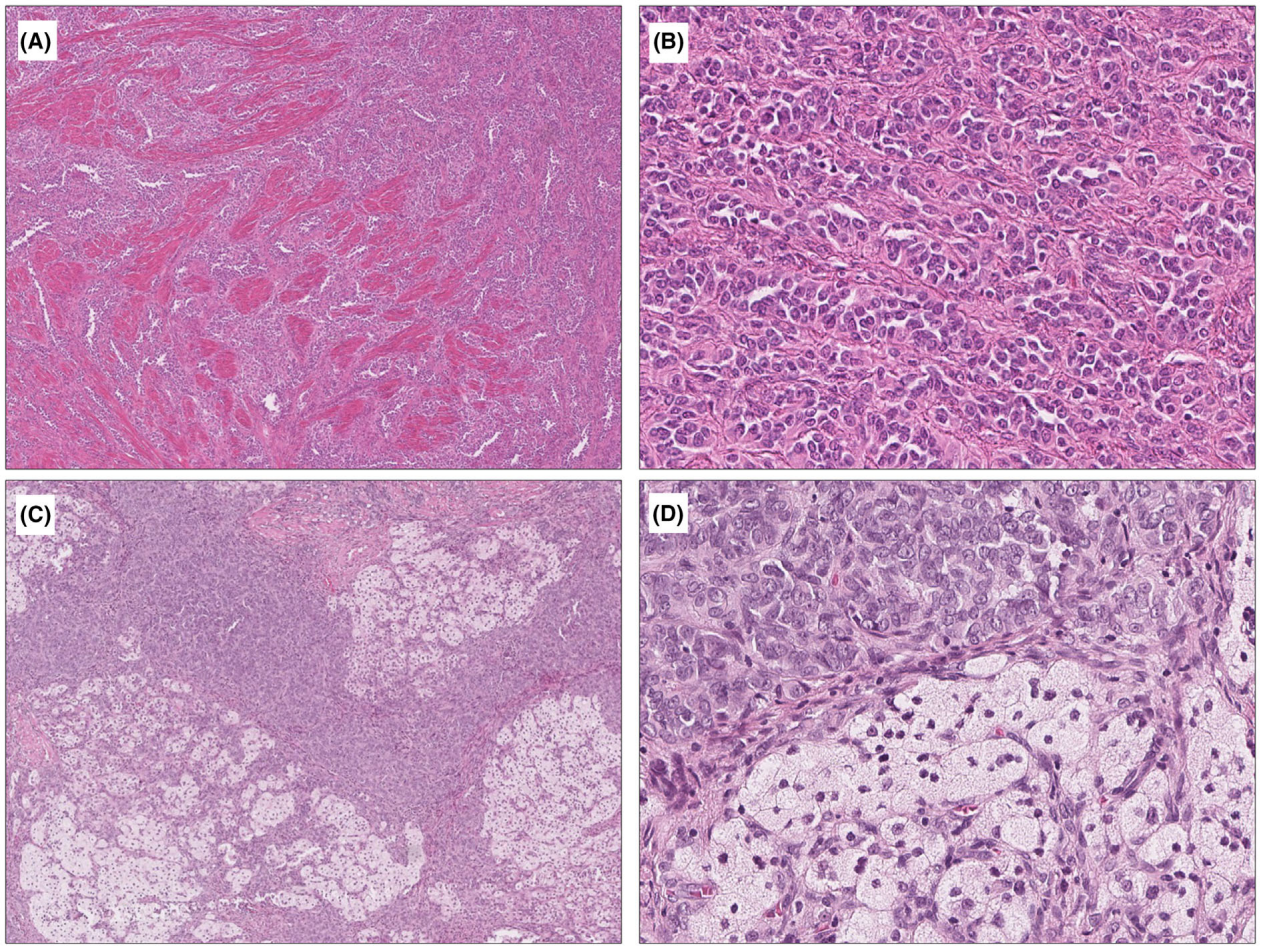
Representative histologic features of two morphologically classic UTROSCTs. (**A**, **B**) UTROSCT (case 17) harbouring an *ESR1::NCOA2* gene fusion shows a sertoliform and trabecular growth pattern with bland cytology and low mitotic activity. (**C**, **D**) UTROSCT (case 9) with unknown fusion status exhibits a predominantly solid and corded architecture with foam cells and hypertrophic smooth muscle bundles.

**Figure 2:**
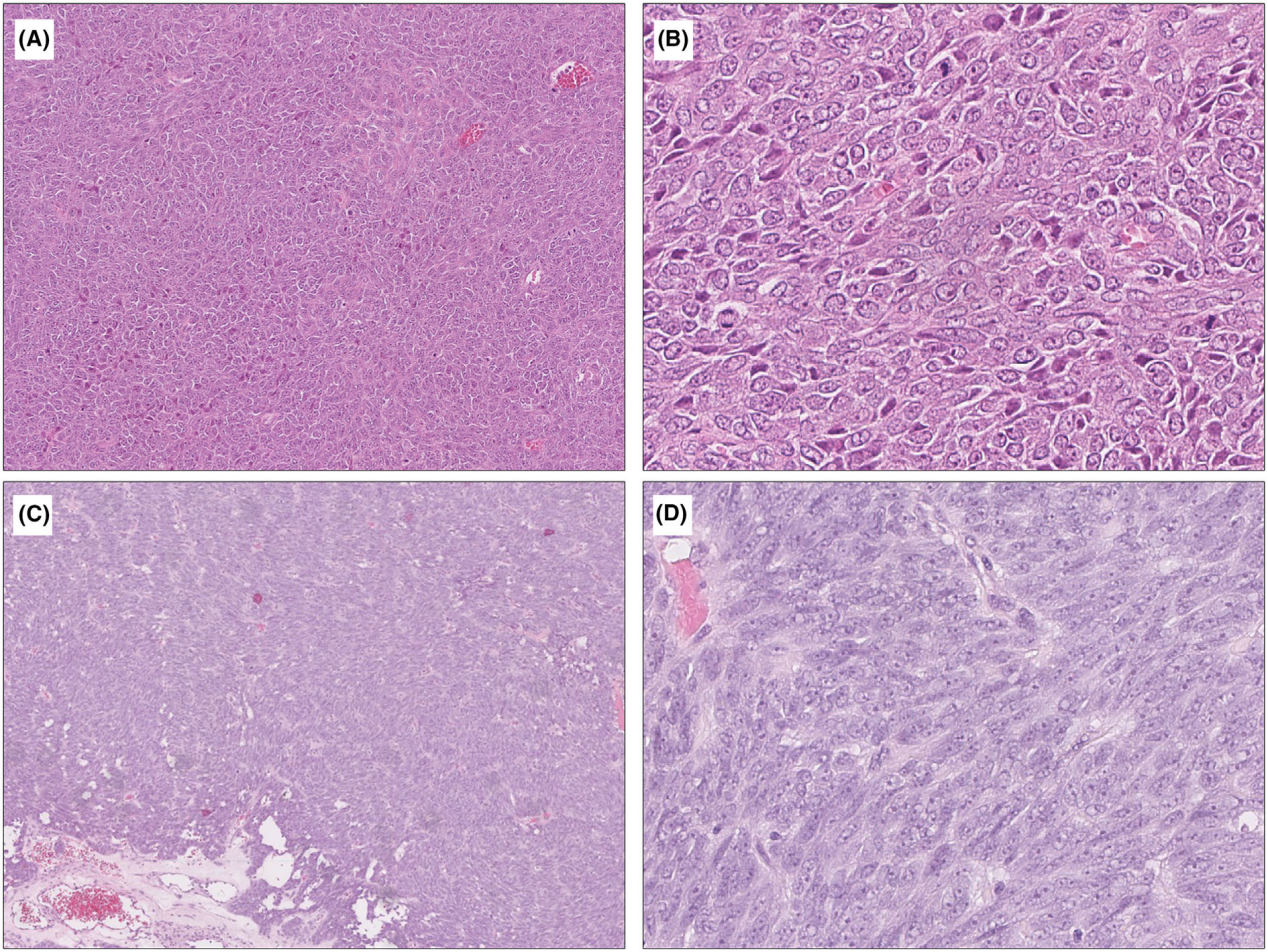
Histologic features of two *GREB1*‐rearranged uterine tumours lacking overt sex cord differentiation. (**A**, **B**) Uterine tumour (case 28) with a *GREB1::NCOA2* gene fusion shows solid, poorly differentiated round to ovoid cells with monomorphic moderate nuclear atypia, central nucleoli and readily identifiable mitotic activity. (**C**, **D**) Uterine tumour (case 25) with a *GREB1::NCOA1* gene fusion displays a solid sheet‐like proliferation of poorly differentiated ovoid to spindle cells. Tumour cells are monomorphic with mild to moderate atypia, central nucleoli and inconspicuous mitoses.

Among the 21 morphologically defined UTROSCTs, only one case (case 16) with *ESR1::NCOA2* fusion displayed a diffuse sheet‐like growth of monomorphic round to ovoid cells with no apparent sex cord differentiation, whereas the remaining tumours (including 6 harbouring *ESR1::NOCA2/3*) displayed mixed patterns of sex cord differentiation that included sertoliform corded, trabecular, tubular and nested growth patterns (Figure 1). A minor component of diffuse pattern was also found in 11 of the 21 cases. Mitotic activity was low in all 21 cases (≤2 MF per 10 HPF/2.37 mm^2^). In comparison, only one *GREB1*‐rearranged UTROSCT displayed prominent sex cord differentiation with a mix of trabecular and corded patterns (case 30), and this case showed focally brisk mitotic activity (Figure 2). The remaining cases showed exclusively or predominantly diffuse sheet‐like growth patterns (Figure 2), with minor sertoliform/trabecular/corded/tubular patterns in 3 cases. Mitotic activity was low in 7 cases (≤4 MF per 10 HPF/2.37 mm^2^) and high in 3 cases (≥9 MF per 10 HPF/2.37 mm^2^).

### GREB1‐Rearranged Uterine Tumours Share a Common DNA Methylation Profile with ESR1‐Rearranged UTROSCT

**Figure 3:**
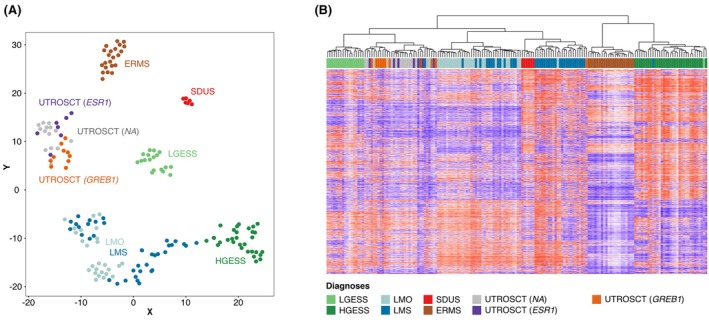
(**A**) 2D representation of pairwise sample correlation using the 10,000 most variable methylated probes by t‐SNE dimensionality reduction. (B) Unsupervised hierarchical clustering (Euclidean ward) of the 10,000 most differentially methylated CpGs. Samples are coloured according to their institutional diagnoses: Low‐grade endometrial stromal sarcomas (LGESS), high‐grade endometrial stromal sarcoma (HGESS), leiomyoma (LMO), leiomyosarcoma (LMS), uterine tumour resembling ovarian sex‐cord tumour (UTROSCT), *SMARCA4*‐deficient uterine sarcoma (SDUS), DICER1‐mutant embryonal rhabdomyosarcoma (ERMS), uterine tumour resembling ovarian sex cord tumour (UTROSCT) with no gene fusion testing available (NA), *ESR1*‐rearranged UTROSCT (UTROSCT *ESR1*) and *GREB1*‐rearranged uterine tumour (UTROSCT *GREB1*).

Genome‐wide DNA methylation analysis of the study cohort identified a distinct cluster for morphologically defined *ESR1*‐rearranged and *GREB1*‐rearranged tumours (Figure 3A,B). This cluster was separate from other clusters of endometrial stromal tumours (LGESS and HGESS) as well as smooth muscle tumours (LMO and LMS), ERMS and SDUS. Interestingly, within the UTROSCT cluster, we observed two subclusters that appeared to loosely correlate with fusion status, despite the presence of one or two outlier cases in each subcluster.

### GREB1‐Rearranged Uterine Tumours Exhibit Greater Genomic Instability Compared to Conventional UTROSCT

**Figure 4:**
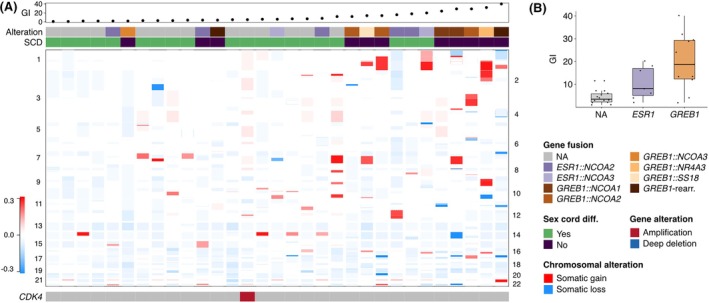
(**A**, **B**) Case‐by‐case copy number profiles of UTROSCT with chromosomal gains depicted in red and losses shown in blue. Above Genomic index (total number of segmental gains or losses^2^/number of involved chromosomes), indicative of genomic complexity, as well as the fusion transcript identified, are annotations as indicated by the figure's legend.

CNV analysis revealed that morphologically defined UTROSCTs, including *ESR1*‐rearranged UTROSCTs, are generally genomically stable tumours (Figure 4A). However, *GREB1*‐rearranged uterine tumours showed a trend for greater genomic instability, with a mean GI of 19.4 (median 19, range 2–40), compared to *ESR1*‐rearranged UTROSCT (*P* = 0.07), which had a mean GI of 10.7 (median 8, range 2–20), and UTROSCT that had not undergone fusion testing (*P* = 0.002), which had a mean GI of 6.31 (median 4, range 1–12) (Figure 4B). One UTROSCT, which had not undergone fusion testing, harboured an amplification of the *CDK4* locus.

## Discussion

Here, we report the global DNA methylation and copy number variation profiles on a series of UTROSCTs, including tumours with *ESR1* rearrangements and *GREB1*‐rearranged uterine tumours, to investigate the epigenetic and molecular profiles between these tumours. With regards to the epigenetic similarities based on their global DNA methylation profile, *GREB1*‐rearranged tumours form part of a cluster that overlaps with *ESR1*‐rearranged UTROSCTs and morphologically defined UTROSCTs, which altogether appear to be distinct from other types of uterine mesenchymal tumours. This addresses an important question regarding tumour nosology. Since our initial reports of *GREB1*‐rearranged uterine tumours/sarcomas with minimal sex cord differentiation, accumulating evidence from subsequent studies has established that *GREB1*‐rearranged uterine tumours fall within the UTROSCT spectrum, given the morphologic overlap. This is now further supported by global DNA methylation analyses, which have demonstrated utility as a robust tool for tumour classification.^16^, ^23^

While global DNA methylation profiles support the notion that *GREB1*‐rearranged uterine tumours are UTROSCTs, there are some major differences between *GREB1*‐rearranged UTROSCTs and *ESR1*‐rearranged UTROSCTs. Genomically, *GREB1*‐rearranged UTROSCTs demonstrate a greater degree of copy number variations compared to *ESR1*‐rearranged UTROSCTs, as reflected by the copy number plots and genomic instability scores. Histologically, we found that *GREB1*‐rearranged UTROSCTs frequently display a diffuse/solid growth pattern with at most focal sex cord differentiation, in contrast to *ESR1*‐rearranged UTROSCTs, in which an exclusively diffuse growth pattern was seen only in one such case. Moreover, mitoses are generally inconspicuous in *ESR1*‐rearranged UTROSCTs, whereas they can be brisk in a subset of *GREB*1‐rearranged UTROSCTs. This is in keeping with previous observations made by us,^12^ as well as the systemic review performed by Maccio *et al*.^15^ Given these differences outlined above (more poorly differentiated/diffuse histology, greater mitotic index and higher degree of genomic instability), it is perhaps not surprising that *GREB1*‐rearranged UTROSCTs appear to be clinically more aggressive than *ESR1*‐rearranged UTROSCTs based on reported cases, with a median disease‐free survival of 95.1 months for *GREB1*‐rearranged UTROSCTs compared to an extrapolated median disease‐free survival of 218 months (as the median survival was not reached in the systemic review) for *ESR1*‐rearranged UTROSCT.^15^ However, future studies are needed to determine whether and how tumour genotype relates to the proposed clinical and pathologic features of malignancy for UTROSCT.^3^

A compelling question that arises is why such differences exist between *GREB1*‐rearranged and *ESR1*‐rearranged UTROSCTs. GREB1 (growth regulation by oestrogen in breast cancer 1) was initially identified as a top oestrogen receptor‐alpha (ESR1) target gene, with its expression induced by ESR1 binding to oestrogen response elements upstream of the *GREB1* promoter in breast cancer.^24^, ^25^ However, in normal uterine endometrium, GREB1 expression can be stimulated by progesterone and GREB1, in turn, facilitates progesterone‐induced gene expression programs. In contrast, in tissues of endometriosis, oestrogen stimulates GREB1 expression, which in turn activates oestrogen‐induced transcriptional programs.^26^ While the precise identity of the UTROSCT progenitor cell remains unknown, these insights raise the intriguing possibility that the level of GREB1 expression in these cells may not be directly proportional to ESR1 expression, given that GREB1 can also be regulated by other sex hormones. This may contribute to the apparent differences in biologic/clinical behaviour and also in the observed difference in age, as *GREB1*‐rearranged UTROSCTs appear to occur in older women compared to *ESR1*‐rearranged UTROSCTs, with an average of 55.7 years compared to 39.9 years, respectively.^15^ An additional mechanistic explanation may lie in the diversity of 3′ fusion partners. *ESR1* fusions in UTROSCTs are largely restricted to *NCOA2* and *NCOA3*, whereas *GREB1* fusions exhibit broader variability, involving partners such as *NCOA1, CTNNB1, SS18* and *NR4A3*.

In summary, we demonstrate through global DNA methylation profiling that *GREB1*‐rearranged uterine tumours share similar DNA methylation profiles with morphologically defined UTROSCTs, including those harbouring *ESR1* rearrangement, indicating that they belong to the UTROSCT family. However, *GREB1*‐rearranged UTROSCTs have a dominant solid growth pattern, are less likely to display conspicuous sex cord differentiation and possess a higher degree of genomic instability in the primary uterine tumours compared to *ESR1*‐rearranged UTROSCTs, which may explain the more aggressive clinical behaviour that has been reported. Our finding indicate that molecular testing may in the future become a diagnostic standard for the proper diagnostic classification of UTROSCT.

## Author Contributions

CHL, YSL and FKFK conceptualized the project. AvD, CK and FKFK coordinated data generation. Data was analysed and visualized by FKFK. CHL, MS and FKFK reviewed histology. CHL, JB, JL, HH, DK, BD, MK, FK and FKFK provided tumours samples and metadata. CHL, YSL and FKFK contributed to the original draft. The final manuscript was reviewed and approved of by all authors.

## Conflict of interest

AvD is the recipient of an Illumina research grant. All other authors state no conflict of interest.

## Data Availability

The data for this study are available upon reasonable request.
